# Effectiveness of Hypopressive Exercises in Women with Pelvic Floor Dysfunction: A Randomised Controlled Trial

**DOI:** 10.3390/jcm9041149

**Published:** 2020-04-17

**Authors:** Beatriz Navarro-Brazález, Virginia Prieto-Gómez, David Prieto-Merino, Beatriz Sánchez-Sánchez, Linda McLean, María Torres-Lacomba

**Affiliations:** 1 Physiotherapy in Women’s Health (FPSM) Research Group. Physiotherapy Department, Faculty of Medicine and Health Sciences, University of Alcalá, Alcalá de Henares, 28805 Madrid, Spain; b.navarro@uah.es (B.N.-B.); v.prieto@uah.es (V.P.-G.); dprieto@ucam.edu (D.P.-M.); maria.torres@uah.es (M.T.-L.); 2 Applied statistical methods in Medical Research Group, Catholic University of Murcia (UCAM), 30107 Murcia, Spain; 3 School of Rehabilitation Sciences, Faculty of Health Sciences, University of Ottawa, Ottawa, ON K1H 8M5, Canada; linda.mclean@uottawa.ca

**Keywords:** hypopressive exercises, adherence, pelvic floor dysfunction, pelvic floor exercise, physiotherapy, quality of life

## Abstract

Hypopressive exercises have emerged as a conservative treatment option for pelvic floor dysfunction (PFD). The aim of this study was to compare the effects of an eight-week hypopressive exercise program to those of an individualized pelvic floor muscle (PFM) training (PFMT) program, and to a combination of both immediately after treatment and at follow-up assessments at 3, 6 and 12 months later. The study was a prospective, single-centre, assessor-blinded, randomised controlled trial. Ninety-four women with PFD were assigned to PFMT (*n* = 32), hypopressive exercises (*n* = 31) or both (*n* = 31). All programs included the same educational component, and instruction about lifestyle interventions and the knack manoeuvre. Primary outcomes were the Pelvic Floor Distress Inventory Short Form (PFDI-20); the Pelvic Floor Impact Questionnaire Short Form (PFIQ-7); PFM strength (manometry and dynamometry) and pelvic floor basal tone (dynamometry). There were no statistically significant differences between groups at baseline, nor after the intervention. Overall, women reduced their symptoms (24.41–30.5 on the PFDI-20); improved their quality of life (14.78–21.49 on the PFIQ-7), improved their PFM strength (8.61–9.32 cmH_2_O on manometry; 106.2–247.7 g on dynamometry), and increased their pelvic floor basal tone (1.8–22.9 g on dynamometry). These data suggest that individual PFMT, hypopressive exercises and a combination of both interventions significantly reduce PFD symptoms, enhance quality of life, and improve PFM strength and basal tone in women with PFD, both in the short and longer term.

## 1. Introduction

Pelvic floor dysfunction (PFD) is associated with urinary incontinence (UI), pelvic organ prolapse (POP), anal incontinence (AI), and sexual dysfunction [1]. These conditions are chronic and are associated with lower quality of life, and reduced physical, social, and mental well-being [2]. Prevalence studies suggest that between 23.7% to 46.2% of women experience at least one PFD [3,4], confirming that these distressing problems are common among females. Related risk factors for PFDs include advanced age, pregnancy, parity, instrumented delivery, high body weight [3,4,5], chronic cough, and the repeated performance of physical exertions that load the pelvic floor [5]. Physical therapy based on pelvic floor muscle training (PFMT) is the first line of conservative treatment for women with UI [6] and early stages of POP [7].

Although there is no standardized protocol for PFMT, one-to-one and closely supervised exercises seem to be the most effective for stress UI [8]. Many physical therapists integrate other training approaches in the management of women with PFD, including abdominal muscle training and postural education, in the belief that such training will mitigate repetitive and/or chronic loading on the pelvic floor [9]. In this context, hypopressive exercises (HEs) have emerged as a treatment option for PFD; currently HEs are widely prescribed for women with PFD in hospitals and in private practice settings in France, Belgium, Spain and Latin American countries [10,11,12,13,14]. In 1980, Caufriez developed a series of thirty-three consecutive HEs which each involved a posture to be performed in different body positions (standing, kneeling, quadruped, sitting and supine), combined with a hypopressive manoeuvre, in which the women performed an expiratory apnea (breath hold at end expiration), while drawing-in their abdomen and opening their rib cage [10,11,12,13,14,15]. The theoretical aim of HEs is to lower intra-abdominal pressure, while concurrently increasing the basal tone of the pelvic floor muscles (PFMs) and deep abdominal muscles without voluntary activation [15].

Recent studies have lent some support to HEs as an intervention for PFDs. There is evidence that HEs produce the neuromuscular activation of PFM and abdominal muscles, which might lead to a beneficial effect on PFM endurance in women with PFD [10]. Evidence also suggests that HEs may increase levator ani muscle thickness [11] and basal tone, and that they reduce UI symptoms [12]. Randomised controlled trials have concluded that HEs do not enhance the beneficial effect of PFMT on the strength nor cross-sectional area of the PFMs in women with POP [13,14]. Nevertheless, in these studies the supervision offered by the study physical therapist was limited, the effect of the therapy on patient-reported symptoms and quality of life quality of life was not assessed, and the treatment effects were only evaluated in the short-term [13,14], immediately following the intervention.

The efficacy of HEs has not been evaluated in women who report a combination of concurrent PFDs [3,4], which is the clinical reality for many gynaecological and physiotherapy services. Furthermore, the longer-term success of HEs has not been evaluated. Lastly, while symptoms return when women abandon PFMT exercises, the adherence of women to home based HEs is not known [16], and the minimum exercise dose required to improve or maintain the outcomes [17] through the HE approach is not known.

Among women with mild PFDs randomised to a PFMT intervention, a HE intervention or a combined PFMT and HE intervention, we hypothesised that (i) those randomised to the combination HE and PFMT intervention would demonstrate greater improvements in patient reported outcomes than those randomised to either intervention alone, (ii) those randomised to the HE intervention (alone or in combination with PFMT) would demonstrate greater increases in PFM basal tone compared to those who performed PFMT alone, (iii) those randomised to PFMT (alone or in combination) would demonstrate greater improvements in PFM strength than those who performed HE alone. Moreover, since women have been shown to be satisfied with HE [11] in addition to achieving an improved sense of well-being [12], we hypothesised that women randomised to the HE intervention would report long-term adherence to the exercise program. Therefore, the aims of the present study were to compare the improvements in women’s self-reported signs and symptoms of PFD and their associated quality of life, as well as differences in PFM strength and tone and adherence to a home exercise program among women with mild PFDs who are randomised to a HEs intervention alone and in combination with PFMT, compared with PFMT alone as a reference group.

## 2. Materials and Methods

We carried out a single-centre, randomised, single-blinded, 3-armed parallel group clinical trial of women referred with PFD to the Physiotherapy in Women’s Health Research Unit of the Alcalá University (Madrid, Spain), between October 2013 and September 2017. The study was registered at ClinicalTrials.gov (NCT02259712) and approved by the Clinical Research Committee of the Principe de Asturias Hospital (OE20/2013). In all cases, participant consent was obtained, and CONSORT guidelines were followed.

### 2.1. Participants

Women who were referred by their general practitioner, urologist, gynaecologist, or midwife to receive pelvi-perineal physiotherapy to manage signs or symptoms of PFD were invited to participate. The inclusion criteria were self-reported signs or symptoms of stress or mixed UI, AI, and/or gynaecologist diagnosis of stage 1 or 2 of POP, according to the POP-Quantification Scheme [1]. The exclusion criteria were: age less than 18 years or over 70 years, pregnancy, pregnancy within the six months prior to referral, underwent physiotherapy for PFD in the previous year, abdominal or pelvic surgery in the previous year, only presenting with symptoms of urge UI, urge faecal incontinence or vaginal pain, concurrent neurological or a psychiatric disease, any medical contraindication to performing therapeutic exercises, not able to attend treatments or follow-up assessments at 3, 6 and 12 months, or the inability to understand and complete the study questionnaires. Participants who agreed to participate provided written informed consent prior to entering the study. Prior to the intervention, each participant was individually assessed (A0).

### 2.2. Randomization and Blinding

After A0, equal numbers of women were randomised to a pelvic floor muscle training group (PFMT-G), a hypopressive exercise group (HE-G) or to a combination group (PFMT+HE-G)). A physical therapist (VPG: PT1), who did not participate in the assessment nor in the intervention, used a computer randomization scheme (EPIDAT v.3.1, Xunta, Galicia, Spain) to allocate participants consecutively to each treatment group. Allocation was not revealed until each participant had completed their baseline assessment, at which time the treating therapist and the participant were informed of their group assignment. The three programs lasted eight weeks, with two visits of 45 min each per week. All programs included an educational component where women learned about PFDs and risk factors, as well as how to perform the knack manoeuvre, contracting their PFMs before and during physical efforts that are known to increase intra-abdominal pressure, such as coughing, sneezing, laughing or jumping. The primary outcomes were the impact of PFD (Pelvic Floor Impact Questionnaire Short Form (PFIQ-7)) and of PFD-related symptoms on quality of life (Pelvic Floor Distress Inventory Short Form (PFDI-20)). The secondary outcomes were PFM strength measured by manometry and dynamometry, PFM basal tone measured by dynamometry, and adherence to the home training program 6 and 12 months after the intervention.

### 2.3. Follow-Up

Initially, four follow-up visits were scheduled: shortly after completing the intervention (A1), and at 3 (A2), 6 (A3) and 12 (A4) months after A1. The appointments were flexible depending on participant availability, and women were called and/or sent a message one week before their scheduled appointment, in order to confirm or change the day/time.

### 2.4. Interventions

The same physical therapist (BNB: PT2), who had more than five years of experience in the physiotherapy management of PFD, delivered all interventions, individually and in person. PT1 and PT2 were the only study members aware of participant’s group allocation.

#### 2.4.1. Pelvic Floor Muscle Training Group (PFMT-G)

Through encouragement, feedback and resistance offered through vaginal palpation in the lithotomy position, participants performed PFM exercises based on components of the PERFECT scheme [18]. At each session, participants were encouraged to achieve ten maximal effort and rapid contractions lasting 1 s each, to maintain an isometric contraction up to 10 s, and to repeat this sequence ten times. Goals were adjusted according to participant progression at every session, and if PT2 considered it appropriate, manual resistance was applied to enhance PFM force. Internal palpation was performed using two fingers inside the vagina and feedback was given based on palpation at the midline (6 o’ clock), the left side (8 o’ clock) and the right side (4 o’ clock), to teach women to train all of their PFMs. At any session, if a woman achieved a score < 3 [19] on levator ani testing (LAT), intravaginal electrical stimulation (using biphasic pulses with frequency = 85 Hz, pulse width = 500 µs and a train: rest period = 4:8, then using biphasic pulses with frequency = 30 Hz, pulse width = 500 µs and a train: rest period of 15:10) was used for 15 min during the session to enhance PFM awareness and contraction. When pain was reported on palpation of the PFMs, local compression was applied to painful points, and local stretching and eccentric PFM exercises were performed [20].

Following these modalities, exercises were performed in the lithotomy position using a manometry probe (Phenix USB 2, Vivaltis, Montpellier, France), interfaced with an IBM compatible computer for biofeedback. The biofeedback system offered different screens to support concentric, isometric, and eccentric PFM exercises; the specific exercises and the timing were adjusted based on women’s capacity and were progressed when appropriate. In women with low PFM contraction awareness (LAT < 3), and in women with large urogenital hiatus, the dynamometry probe, which could be opened to provide tactile feedback, was used instead of manometry. Women also progressed from manometry to dynamometry once they were capable of generating pressure while performing the exercises, as more resistance could be provided by opening the arms of the dynamometer. If women progressed enough, the last two biofeedback sessions were conducted in a more functional standing position.

After each treatment session, women were instructed to perform one to three sets of 5 to 10 repetitions PFM exercises daily at home, in supine, sitting or standing position, based on their PERFECT evaluation, daily, between 1 and 3 times per day.

#### 2.4.2. Hypopressive Exercise Group (HE-G)

Women were instructed on HEs described as Hypopressive Abdominal Gymnastics by Caufriez [15]. First, participants learned how to perform the “hypopressive manoeuvre”, which consisted of exhaling to their expiratory reserve volume, then holding their breath (apnea), and expanding their rib cage, to draw their abdominal wall inward and cranially without inhalation [10]. Women were asked to sustain the apnea and rib-cage expansion for approximately 10 s before resuming their normal breathing. When the participants were capable of performing this manoeuvre in supine, standing and sitting positions, they were then instructed on the series of “hypopressive postures”. These postures are described in standing, kneeling, four-point kneeling, sitting and supine positions, using a variety of upper and lower limb positions [10,15]. While holding the hypopressive posture, the hypopressive manoeuvre was repeated three times, with a rest breath between repetitions; the entire sequence being referred to as a HE [10]. Each HE was repeated three times with rest between exercises. Between 5 and 10 HEs were performed within each session based on the participant’s mastery of the exercises and readiness to progress through the 33 HEs described by Caufriez. The participants were consistently instructed during each exercise not to voluntarily contract their PFMs nor their abdominal muscles.

Women allocated to this group received feedback on how to perform PFM contractions during the vaginal palpation assessment performed with PT3, and were instructed to use this contraction prior to and during tasks that increase intra-abdominal pressure (the knack), but were asked not to perform any specific PFM exercises.

At the end of each intervention session, women were asked to perform three different HEs at home, the selection of which was based on participants choice and the PT2 professional opinion of PT2, the latter based on observing the participant’s mastery of the exercise. Each HE was to be repeated three times per set, and participants were asked to perform between 1 and 3 sets per day.

#### 2.4.3. Combination Training Group (PFMT+HE-G)

Women randomised to PFMT+HE-G performed both PFM exercises and HEs. These participants received the same PFMT intervention as the PFMT group, and were additionally trained to perform the hypopressive manoeuvre and learned five HEs: two postures in supine, one on four-point kneeling, and two in standing. To limit performance bias, the duration of the treatment sessions remained the same in all three groups, therefore the combination group spent half of each session on PFMT and half on HEs. Nonetheless, PFM contractions were never combined with HEs.

After each intervention session, participants were asked to exercise at home, following the exercise prescriptions described for each group, alternating between PFMT and HE between days.

#### 2.4.4. Educational Strategy (All Groups)

The educational strategy consisted of instruction, using printed materials and 3-dimensional anatomical models, on the anatomy of the pelvic floor and the physiology of the pelvic organs. Women were advised to minimize their risk factors by not gaining weight or smoking, limiting caffeine intake, optimizing nutritional intake to limit constipation, and avoiding weightlifting and other high impact sports. They were also instructed on proper toileting habits to avoid straining the pelvic floor and were taught to use the knack manoeuvre before and during tasks that increase intra-abdominal pressure.

### 2.5. Physiotherapy Assessment

A different physical therapist, who specialized in women’s health (MTL: PT3), and who remained blinded to participant group allocation, performed all baseline and follow-up assessments. Participants were instructed not to reveal their allocation to PT3.

At the baseline assessment (A0), personal data including age, body mass index, physical activity, and clinical and obstetric history were collected. At A0 and all other assessments, the following outcomes were collected by PT3:

(1) The PFIQ-7 Spanish version [21] consists of three scales of seven questions, each taken from the Urinary Incontinence Impact Questionnaire, the Pelvic Organ Prolapse Impact Questionnaire, and the Colorectal-Anal Impact Questionnaire. The three scales are scored from 0 (least impact) to 100 (greatest impact) and an overall summary score (0 to 300) describes the impact of PDF on day to day activities.

(2) The PFDI-20 Spanish version [21] measures both pelvic floor symptoms and the degree of bother and distress associated with those symptoms. The PFDI-20 includes 20 questions and three scales. Each of the three scales is scored from 0 (least distress) to 100 (greatest distress), again with an overall score ranging from 0–300 and higher scores indicating lower quality of life. The three scales include questions taken from the Urogenital Distress Inventory—6 questions, the Pelvic Organ Prolapse Distress Inventory—6 questions, and the Colorectal-Anal Distress Inventory—8 questions.

(3) PFM function was assessed using three different measurement instruments. Vaginal palpation was used to score PFM function using the LAT [19,22], which is a subjective evaluation ranging from 0 to 5, based on a combination of muscle strength and endurance. PFM strength was then objectively measured with manometry (Peritron, Melbourne, Australia) and intravaginal dynamometry (Pelvimètre Phenix, Montpellier, France). Three maximum effort PFM contractions were performed using each device and the mean peak value of the three trials was retained for analysis. For the manometry, an air-filled probe was used, and for dynamometry, a two-armed speculum was used with the arms oriented to open in the mid-sagittal plane, but retained in the closed position. Both vaginal probes were connected to a Phenix USB2 biofeedback system (Vivaltis, Montpellier, France), interfaced with an IBM compatible computer, and protected by latex or polyethylene covers. Basal tone was measured (in g) by dynamometry, before measurements of PFM strength were made. The dynamometry probe was inserted into the vagina with the arms closed, and passive force was measured as women were asked to completely relax their PFMs [22].

During the assessment of PFM strength, contraction quality data were recorded based on visual inspection, including simultaneous contraction of the gluteus maximus, hip adductor and/or abdominal muscles, and breath holding.

Exercise adherence was evaluated by PT3, who asked participants at A3 and A4 if they were doing their home exercises, and, if so, how many times per week. She also asked participants if they had incorporated the knack manoeuvre into their daily activities.

### 2.6. Data Analysis

We estimated that with a sample size of 33 individuals in each arm we would have 80% power to detect a between-group difference of 10 points in the change of quality of life score (PFDI-20), assuming an expected average change in the reference group (PFMT group) of 25 points with a standard deviation of 20 points, as was observed in a previous study [13].

We summarized categorical variables with proportions and continuous variables with means and standard deviations (SD). To estimate the average change from baseline to subsequent visits (V1, V2, V3 and V4) for each of the continuous outcomes (PFIQ-7, PFDI-20, PFM strength, and PFM passive resistance), we generated separate linear regression models for each visit, adjusting for the values of that outcome at A0, as indicated in Equation (1):

Y_t_ = B_0_ + B_1_Group + B_2_Y_0_ + B_3_(Group*Y_0_) (1)

where Y_t_ is the variable of interest at follow-up visit t and Y_0_ is the baseline value of that variable (both centred at baseline mean E(Y_0_)). Group is a categorical variable. The coefficient B_0_ captures the average difference between visits 0 and t in the reference group. By changing the reference group and re-estimating the model, we can estimate the effects in each group.

To compare interventions, we tested whether changes in outcomes across the four follow-up visits were different between groups. We analysed data from all visits together in one repeated-measures linear regression model, including individual as a random factor (to account for repeated measures) and visit and intervention group as fixed factors, while the baseline value was included as a covariate to adjust for regression to the mean. The model also included the interaction between group and baseline values (see Equation (2)):

Y_t_ = B_0_ + B_1_Group + B_2_Y_0_ + B_3_(Group*Y_0_) + B_4_Visit | random(id) (2)

Visit and Group were included as categorical variables, with the PFMT-G intervention group as the reference. Thus, there were two separate B_1_ coefficients that capture the average differences between the HE-G and PFMT+HE-G groups and the PFMT-G group across all three follow up visits. This model has more power than the previous model in equation-1 to detect differences between groups, because it considers the effect of time (the visit) and the possible regression to the mean from baseline values (allowing for differential effects of regression to the mean in the different groups).

The adherence to exercises at A3 and A4 was studied by analysing two binary outcomes (“does exercises at home regularly” and “does the knack”). For each outcome, we used a logistic regression model with trial arm as a categorical variable and PFMT-G as the reference group. The variable LAT was also analysed as binary, because its limited scale (0 to 5) clearly broke the assumptions of normally needed for the linear models above. We compared the proportions of patients with LAT = 5 at A4 between the three treatment groups, using a logistic regression model as explained above for the analysis of adherence to exercises. An α = 0.05 was used for all tests.

## 3. Results

**Figure 1:**
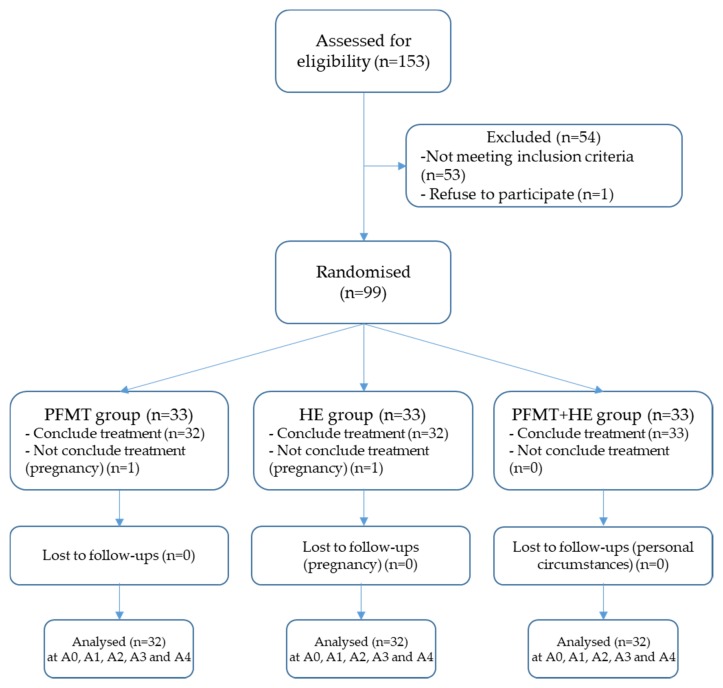
Flow diagram of participants.

In total, 99 women were included in the study. Two women could not complete the intervention because they became pregnant and three other participants did not complete the follow-up and had no final assessment. None of these five participants’ data were included in the final analysis. In the end, 94 participants completed the intervention and follow-up visits, *n* = 32 in the PFMT-G, *n* = 31 in the HE-G, and *n* = 31 in the PFMT+HE-G (see flow diagram in Figure 1). Clinical and demographic characteristics at baseline are shown in Table 1.

**Figure 2:**
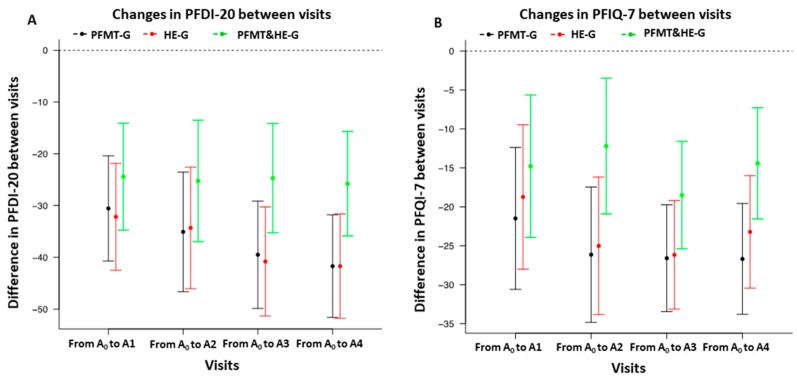
(**A**): changes in PFDI-20: Pelvic floor Distress Inventory Short Form outcome from baseline; (**B**): changes in PFIQ-7: Pelvic Floor Impact Questionnaire Short Form outcome from baseline.

Table 2 shows the average differences from baseline to each follow-up visit, for each outcome of interest and for each intervention group. The mean differences were estimated after adjusting for baseline values, as described in Equation (1). For most of the outcomes at most follow-up visits, the 95% confidence intervals did not include “0”, indicating 95% confidence that a true change in that outcome occurred relative to baseline. The intervals that included the null are highlighted in bold font. For the main quality of life outcomes (PFDI-20 and PFIQ-7) the confidence intervals did not include 0 (see Figure 2).

**Figure 3:**
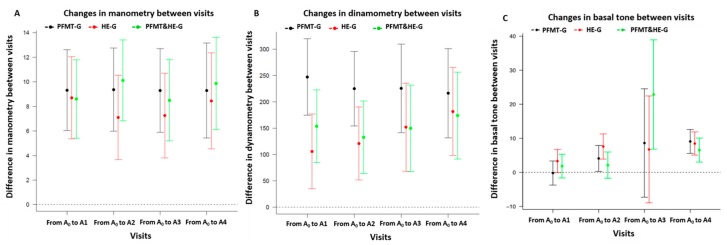
(**3**): changes in pelvic floor muscle strength measured by manometry outcome from baseline; (**3**): changes in pelvic floor muscle strength measured by dynamometry outcome from baseline; (**3**): changes in basal tone outcome from baseline.

Regarding PFM function outcomes, women in all three groups improved their PFM strength measured both by manometry and dynamometry (Table 2); whereas tone increased at A2 in PFMT-G and HE-G, at A3 in PFMT+HE-G and at A4 in all groups (Figure 3). Only two patients had LAT = 5 at baseline, while 44 patients (47%) had LAT = 5 at visit A4, with a similar proportion in the three trial arms (50% in the PFMT group, and 45% in each of the two other groups). These differences between the groups are not statistically significant (*p*-value from a X2 test of homogeneity is 0.91). 

Table 3 shows the coefficients for the average difference between groups across all three visits for the different outcomes (estimated using Equation (2)). Only three variables were significantly different (*p* < 0.05) and the primary outcomes (PFDI-20 and PFIQ-7) did not show significant differences.

Some adverse effects were found in the three intervention groups. One woman in the PFMT+HE-G reported that the exercises exacerbated back pain, so the exercises were adapted to lower the intensity and number of sets, and five women who were instructed in HEs, needed the postures adapted because they could not do them correctly or they caused pain when performing them as instructed (which was as described by Caufriez).

Adherence to the home exercises reported in A3 was similar across the groups; PFMT-G had 23 (71.9%) adherence, HE-G had 19 (61.3%) adherence and PFMT+HE-G had 21 (67.7%) adherence, in terms of exercising weekly at home. By A4 adherence fell below 60% (56.3%, 54.8%, and 48.4% in PFMT-G, HE-G and PFMT+HE-G respectively) in all three treatment groups, with no significant differences between groups. The knack manoeuvre was incorporated into activities of daily life in approximately 85% of participants, with no significant differences found between groups. Again, some reduction in the performance of the knack was observed at A4 (68.8%, 83.9%, and 80.6% in PFMT-G, HE-G and PFMT+HE-G respectively), again with no significant differences between groups.

## 4. Discussion

To our knowledge, this is the first study to test the efficacy of HEs alone, or in combination with PFMT, against PFMT in order to determine the efficacy of these exercises, in relation to the gold standard for conservative intervention. The findings from this study suggest that, when delivered in conjunction with education and advice to perform the knack manoeuvre, a physiotherapy treatment focused on PFMT, HEs or a combination of PFMT+HEs all reduce PFD symptoms, improve condition specific quality of life, and achieve an enhancement in PFM function. As the goal of the treatment for PFD is to maintain improvements over the long term, we investigated both the maintenance of improvements after the end of the eight-week supervised protocol, as well as adherence to the home exercise program after the intervention was discontinued. We found that improvements were maintained, and adherence to the exercises and to the knack was high, as 97 women completed the intervention, 94 participants attended the four follow-ups, and 50 women continued to practice the exercises at home, 12 months after the treatment ended.

While no significant differences were found between the study groups, all groups improved in terms of their symptoms; some limitations in terms of the interpretation of these findings must be considered. Firstly, the three groups received the same lifestyle advice, which has itself demonstrated effectiveness in reducing symptoms associated with mild PFD [23]. Second, all women were instructed on the knack manoeuvre, and its value when incorporating it into activities where rises in intra-abdominal pressure are experienced. This approach has also demonstrated effectiveness as a means of reducing or preventing urine leakage [24]. Indeed, with the high adherence to “the knack”, the improvements in PFM strength may have been the result of a training effect induced by performing this manoeuvre. Nevertheless, our intervention approaches were designed in accordance with current physiotherapy practice, where therapeutic exercise is accompanied by education and advice.

The treatment effect was determined as the difference in the mean change in PFDI-20 and PFIQ-7 scores among groups. Barber et al. [25] considered a change of 45 points or more in the summary score of the PFDI-20 and 36 points or more in the PFIQ-7 to be the minimal clinically important difference in women undergoing surgery for PFD. Based on our results, participants improved between 24.4 and 41.7 points in the PFDI-20, and between 14.4 and 26.7 in the PFIQ-7, and as such, the changes seen with the interventions as delivered may not have been clinically important. These small differences may be due to our sample, including women with mild PFD in whom surgery was not indicated, which may have resulted in lower PFDI-20 and PFIQ-7 scores at baseline. In fact, the changes observed in the current study were higher than those observed in the PFMT group of Wiegersma et al. [26], where an improvement of 11 points on the PFDI-20 and 2.6 points on the PFIQ-7 was found in women with mild POP.

Consistent with our results, previous studies have found that supervised PFMT, including biofeedback and electrical stimulation with voluntary PFM contractions, successfully improved the symptoms of UI [27] and mild POP [28]. PFMT without feedback devices has also demonstrated improvements in PFM strength [28] and hypertrophy of the urethral sphincter [27]. Based on our results, although the PFM strength increase was higher in the PFMT group, there were no statistically significant differences between the strength increase in the PFMT-G and that observed in the HE-G and the combined exercise group, as we hypothesised. The addition of HE to a PFMT protocol was previously tested in women with POP-Q stage 2 [13,14] and there were no differences between the PFMT group, and the PFMT+HE group; while PFMT was superior in terms of improving PFM endurance [13]. However, in [13,14], participants only received three supervised session, and PFM contractions were added to HEs, while in our study, all participants received sixteen individual sessions and HEs were trained according to their original description [10,11,12,13,14,15], that is, without voluntary contraction of the PFMs.

The efficacy of a HE training protocol alone or in addition to PFMT has not previously been demonstrated [29], in terms of signs, symptoms, quality of life, PFM strength or PFM basal tone in women with PFD. Our study suggests that a physiotherapy treatment based on HE may be as effective as a PFMT protocol. Hung et al. [9] proposed a treatment based on a coordinated retraining of the diaphragm, the deep abdominal muscles and the PFMs, and demonstrated positive results in women suffering from stress and mixed UI. Although the contraction of the deep abdominal muscles has been identified to occur synergistically to enhance PFM contraction in healthy women [30], it is necessary to measure what happens in women with PFD. When an abdominal muscle contraction causes an increase in intra-abdominal pressure, without an effective PFM activation, a downward movement of the bladder and pelvic floor have been noted [31]. This consequence may strain the connective tissue and/or enlarge the urogenital hiatus. Thus, HEs may be a safe alternative to training the PFMs and abdominal muscles [10], and abdominal muscles while protecting the pelvic floor.

In HEs, the abdominal muscles are not voluntarily activated, and the diaphragm is thought to maintain a shortened position (i.e., end expiration), which theoretically reduces intra-abdominal pressure. While this mechanism has not been demonstrated empirically, in a study using transabdominal ultrasound, it was demonstrated that HEs elevate the PFMs without a direct contraction command [32], and that both the size and the strength of the PFMs were strengthened in the postpartum period in women who performed them for two months, in small groups, for 45 to 60 min, once per week [11]. Furthermore, it was recently observed that HEs recruit the PFMs to 74.4%–86.5% of their maximum electromyography activation and generate vaginal closure forces between 51.2%–55.7% of the maximum observed during voluntary contraction [10]. Thus, HEs may provide a PFM training stimulus without voluntary PFM contraction.

However, as noted above, to perform the knack manoeuvre correctly, the PFMs must contract voluntarily, which may mean that the HEs were not the stimulus underlying the improvements in PFM strength, nor the improved symptoms in women with PFD randomized to the HE-G int his study. In this study sample, 7 (7.4%), women demonstrated no motion of the PFMs while trying to contract them (LAT = 0), while 23 (24.6%) achieved a flicker or weak PFM action with no cranial movement of the PFMs (LAT < 3), which suggests that overall, 32% of our sample did not know how to contract their PFMs correctly. All participants were provided feedback through manual palpation on how to contract their PFMs, which may have resulted in positive feedback [33], which is a predictor of success with exercise interventions [34]. Therefore, the success of our interventions, apart from the three exercise protocols, might have been due to the instructions on the knack manoeuvre, feedback provided during intra-vaginal assessments, the intensive protocol, and the individualized and close follow-up.

Soriano et al. [12] did not identify any negative events associated with HEs, although blood pressure was monitored, and participants were asked about respiratory or cardiovascular complications during the treatment. In our study, the appearance of low back pain was identified in five patients, which was resolved in all cases by simplifying the HE postures.

The initial adherence with the treatment was high, where 94 out of 99 participants completed the intensive 2-month intervention and returned for the 12-month follow-up. The maintenance of achieved improvements after the discontinuation of physiotherapy treatment is a main goal [16]. Our results corroborate those from Borello-France et al. [17], who described that the improvements reached after an intensive PFMT program in women with stress UI were sustained over a six-month follow-up period. Our adherence rate was high in all three groups, where 60%–70% of women reported that they continued PFM training at least weekly, and 85% claimed to perform the knack manoeuvre when necessary. In long-term studies 1 to 5 years after an intensive physiotherapy treatment, PFM strength and reduced symptoms were maintained, and were attributed to the fact that 70% of women continued to exercise their PFMs at least once a week [35]. However, it has been reported that 15 years after PFMT intervention, adherence drops to 28% and benefits are not maintained [36]. Our positive results in a medium-term follow-up may have been attributed to the intensive, positive result of exercise and the continuation of the knack during tasks that increase intra-abdominal pressure [37]. A limitation of this study was that adherence was self-reported by the participants to the study physiotherapist, and was reported dichotomously. Women may indeed have performed exercises, but at a much lower frequency and intensity than prescribed, or may have reported that they were still performing the exercises because that is what they thought the physiotherapist would want to hear. In order to improve adherence, it would be required to address in depth the difficulties and the reasons that generate that some patients continue, and others do not persist in practicing home exercises.

A final limitation to consider in interpreting the study findings is that the PFMT-G and PFMT+HE-G groups were more familiar with the instruments used to assess PFM strength and basal tone at the follow up assessment than the HE-G, since they were used in the treatment protocol. However, this did not appear to be a problem, since PFM strength and tone were deemed to have improved in all group, and regardless, the primary outcome variables were related to symptoms and quality of life.

## 5. Conclusions

Multi-modal physiotherapy treatments based on PFMT, HE, or both, and all including an educational program, the knack manoeuvre and home exercises, significantly reduced PFD symptoms, enhanced quality of life and improved PFM strength and tone among women with different combinations of mild symptoms of pelvic floor dysfunction, including stress or mixed UI, AI and/or POP (stages I–II). Improvements were sustained up to 12 months later, where 53% of participants reported continued adherence to the home exercises and 78% continued to incorporate the knack manoeuvre into their activities of daily life.

## Figures and Tables

**Table 1:** Baseline characteristics of women.

Characteristics	PFMT Group (*n* = 32)	HE Group (*n* = 31)	PFMT+HE Group (*n* = 31)	Total Sample (*n* = 94)
Age years, (SD)	48 (12)	48 (8)	46 (8)	47 (10)
BMI kg/m^2^, (SD)	24.39 (4.77)	24.63 (3.72)	26.21 (4.73)	25.07 (4.47)
Menopause, *n* (%)	14 (44%)	10 (32%)	10 (32%)	34 (36%)
Parity, (SD)	2 (1)	2 (1)	2 (1)	2 (1)
Smoking, *n* (%)	2 (6.3%)	8 (25.8%)	7 (22.6%)	17 (18.1%)
High blood pressure, *n* (%)	2 (6.3%)	2 (6.5%)	2 (6.5%)	6 (6.4%)
Depression, *n* (%)	4 (12.5%)	3 (9.7%)	7 (22.6%)	14 (14.9%)
Respiratory disease, *n* (%)	3 (9.4%)	8 (25.8%)	3 (9.7%)	14 (14.9%)
Constipation, *n* (%)	10 (31.3%)	14 (45.2%)	10 (32.3%)	34 (36.2%)
Physical activity, *n* (%)	24 (75%)	24 (77.4%)	21 (67.7%)	69 (73.4%)
Pelvic floor dysfunction				94 (100%)
UI, *n* (%)	27 (84.4%)	26 (83.9%)	26 (83.9%)	79 (84.0%)
AI, *n* (%)	13 (56.3%)	17 (54.8%)	9 (29.0%)	44 (46.8%)
POP, *n* (%)	13 (40.6%)	11 (35.5%)	19 (61.3%)	43 (45.7%)
Previous PF surgery, *n* (%)	3 (9.4%)	4 (12.9%)	1 (3.4%)	8 (8.5%)
PFDI-20 total, (DE)	71.71 (45.22)	70.20 (35.23)	69.19 (51.62)	70.38 (44.08)
POPDI, (SD)	18.49 (14.58)	17.61 (14.34)	22.45 (21.05)	19.50 (16.87)
CRADI, (SD)	16.51 (18.26)	20.47 (14.57)	14.22 (12.07)	17.06 (15.27)
UDI, (SD)	36.72 (21.93)	32.12 (21.44)	32.53 (25.22)	33.82 (22.76)
PFIQ-7 V0 total, (SD)	45.39 (43.71)	32.26 (31.36)	35.48 (28.57)	37.79 (35.34)
POPIQ, (SD)	11.16 (16.96)	7.68 (12.94)	9.37 (13.72)	9.42 (14.58)
CRAIQ, (SD)	11.31 (18.09)	4.15 (9.18)	4.91 (8.65)	6.84 (13.07)
UIQ, (SD)	22.92 (19.52)	20.43 (19.87)	21.20 (19.02)	21.53 (19.29)
LAT grade				
0, *n* (%)	3 (9.4%)	1 (3.2%)	3 (9.7%)	7 (7.4%)
1, *n* (%)	4 (12.5%)	2 (6.5%)	6 (19.4%)	12 (12.8%)
2, *n* (%)	5 (15.6%)	2 (6.5%)	4 (12.9%)	11 (11.7%)
3, *n* (%)	15 (46.9%)	15 (48.4%)	11 (35.5%)	41 (43.6%)
4, *n* (%)	4(12.5%)	10 (32.3%)	7 (22.6%)	21 (22.3%)
5, *n* (%)	1 (3.1%)	1 (3.2%)	0 (0.0%)	2 (2.1%)
Manometry cmH_2_O, (SD)	16.51 (14.19)	22.87 (15.92)	18.0 (13.86)	19.11 (14.76)
Dynamometry g, (SD)	239.16 (216.10)	370.20 (307.03)	268.74 (216.63)	293.55 (254.57)
Basal tone g, (SD)	206.67 (17.80)	204.13 (13.70)	200.47 (15.73)	203.76 (15.84)
Nearby muscles contraction, *n* (%)	27 (84.4%)	23 (74.2%)	25 (77.4%)	74 (78.7%)
Apnea during PFM contraction, *n* (%)	27 (84.4%)	17 (54.8%)	25 (80.6%)	69 (73.4%)

PFMT: Pelvic floor muscle training; HE: Hypopressive exercises; PFMT+HE: Pelvic floor muscle training and hypopressive exercises; *n*: Number; SD: Standard deviation; BMI: Body mass index; UI: Urinary incontinence; AI: Anal incontinence; POP: Pelvic organ prolapse; PF: Pelvic floor; PFDI-20: Pelvic floor Distress Inventory Short Form; POPDI: Pelvic organ prolapse distress inventory; UDI: Urinary distress inventory; CRADI: Colo-rectal-anal distress inventory; PFIQ-7: Pelvic Floor Impact Questionnaire Short Form; POPIQ: Prolapse impact questionnaire; CRAIQ: Colo-rectal-anal impact questionnaire; UIQ: Urinary impact questionnaire; LAT: Levator Ani Testing; PFM: Pelvic floor muscles. There were no found significant differences between groups.

**Table 2:** Average changes for each outcome between baseline measurement and different periods (adjusted by baseline).

Variable	To	PFMT Group		HE Group		PFMT+HE Group	
		Mean	95%CI	Mean	95%CI	Mean	95%CI
PFDI-20	A1	−30.55	(−40.70 to −20.39)	−32.17	(−42.48 to −21.86)	−24.41	(−34.72 to −14.09)
PFDI-20	A2	−35.07	(−46.63 to −23.52)	−34.30	(−46.03 to −22.57)	−25.24	(−36.98 to −13.50)
PFDI-20	A3	−39.49	(-49.86 to −29.11)	−40.80	(−51.34 to −30.27)	−24.71	(−35.25 to −14.17)
PFDI-20	A4	−41.70	(−51.61 to −31.78)	−41.69	(−51.76 to −31.62)	−25.77	(−35.85 to −15.69)
POPDI	A1	−7.95	(−11.83 to −4.07)	−9.07	(−13.04 to −5.10)	−5.82	(−9.79 to −1.84)
POPDI	A2	−8.24	(−12.84 to −3.63)	−8.49	(−13.20 to −3.78)	−4.75	(−9.46 to −0.03)
POPDI	A3	−9.44	(−13.22 to −5.66)	−10.84	(−14.71 to −6.98)	−6.77	(−10.64 to −2.90)
POPDI	A4	−13.11	(−16.94 to −9.29)	−10.58	(−14.49 to −6.67)	−6.10	(−10.02 to −2.18)
CRADI	A1	−5.91	(−9.42 to −2.40)	−7.28	(−10.95 to −3.62)	**−3.21**	**(−6.87 to 0.46)**
CRADI	A2	−7.54	(−11.58 to −3.49)	−6.53	(−10.75 to −2.30)	−5.50	(−9.73 to −1.28)
CRADI	A3	−9.44	(−12.74 to −6.15)	−8.72	(−12.16 to −5.28)	−5.76	(−9.21 to −2.32)
CRADI	A4	−8.17	(−11.66 to −4.67)	−9.57	(−13.22 to −5.92)	−4.21	(−7.86 to −0.56)
UDI	A1	−15.83	(−21.45 to −10.22)	−15.74	(−21.41 to −10.06)	−15.11	(−20.77 to −9.44)
UDI	A2	−21.06	(−26.44 to −15.69)	−17.03	(−22.46 to −11.60)	−14.62	(−20.04 to −9.20)
UDI	A3	−20.30	(−25.82 to −14.77)	−21.21	(−26.80 to −15.63)	−12.99	(−18.56 to −7.42)
UDI	A4	−20.57	(−25.29 to −15.84)	−21.44	(−26.21 to −16.67)	−15.77	(−20.54 to −11.01)
PFIQ-7	A1	−21.49	(−30.60 to −12.38)	−18.73	(−28.00 to −9.47)	−14.78	(−23.93 to −5.64)
PFIQ-7	A2	−26.14	(−34.83 to −17.45)	−25.01	(−33.84 to −16.18)	−12.21	(−20.93 to −3.48)
PFIQ-7	A3	−26.6	(−33.46 to −19.74)	−26.17	(−33.14 to −19.19)	−18.50	(−25.39 to −11.62)
PFIQ-7	A4	−26.69	(−33.79 to −19.58)	−23.22	(−30.44 to −16.00)	−14.41	(−21.55 to −7.28)
POPIQ	A1	−5.57	(−9.86 to −1.27)	−5.01	(−9.39 to −0.63)	**−2.96**	**(−7.30 to 1.38)**
POPIQ	A2	−7.92	(−11.94 to −3.90)	−6.29	(−10.39 to −2.19)	**−2.03**	**(−6.09 to 2.04)**
POPIQ	A3	−7.30	(−10.15 to −4.45)	−6.16	(−9.07 to −3.26)	−4.03	(−6.91 to −1.15)
POPIQ	A4	−6.88	(−9.68 to −4.09)	−6.02	(−8.87 to −3.17)	**−2.02**	**(−4.84 to 0.81)**
CRAIQ	A1	−5.17	(−8.49 to −1.86)	−3.98	(−7.39 to −0.57)	**−3.05**	**(−6.39 to 0.30)**
CRAIQ	A2	−5.36	(−9.11 to −1.61)	−4.49	(−8.34 to −0.63)	**−0.14**	**(−3.93 to 3.65)**
CRAIQ	A3	−5.65	(−7.78 to −3.53)	−4.81	(−7.00 to −2.62)	−2.53	(−4.68 to −0.38)
CRAIQ	A4	−6.01	(−8.05 to −3.97)	−2.35	(−4.45 to −0.26)	**−1.04**	**(−3.10 to 1.02)**
UIQ	A1	−11.05	(−15.13 to −6.97)	−10.9	(−15.04 to −6.75)	−10.13	(−14.27 to −6.00)
UIQ	A2	−13.45	(−17.19 to −9.70)	−14.31	(−18.11 to −10.51)	−11.18	(−14.97 to −7.39)
UIQ	A3	−13.70	(−17.30 to −10.10)	−15.00	(−18.65 to −11.35)	−12.48	(−16.13 to −8.83)
UIQ	A4	−13.40	(−17.61 to −9.19)	−14.75	(−19.02 to −10.48)	−10.55	(−14.81 to −6.28)
Manometry	A1	9.32	(6.03 to 12.61)	8.70	(5.37 to 12.03)	8.61	(5.41 to 11.80)
Manometry	A2	9.36	(5.97 to 12.75)	7.10	(3.67 to 10.53)	10.12	(6.83 to 13.41)
Manometry	A3	9.30	(5.88 to 12.71)	7.25	(3.80 to 10.70)	8.50	(5.19 to 11.82)
Manometry	A4	9.31	(5.44 to 13.18)	8.46	(4.55 to 12.37)	9.88	(6.12 to 13.63)
Dynamometry	A1	247.68	(175.12 to 320.23)	106.18	(35.12 to 177.23)	153.84	(85.02 to 222.65)
Dynamometry	A2	225.28	(154.72 to 295.84)	121.12	(51.74 to 190.49)	132.80	(63.98 to 201.62)
Dynamometry	A3	225.80	(141.85 to 309.76)	152.09	(68.36 to 235.83)	149.98	(68.10 to 231.87)
Dynamometry	A4	216.68	(132.01 to 301.34)	181.80	(98.56 to 265.05)	174.22	(91.65 to 256.80)
Basal tone	A1	**−0.2**	**(−3.74 to 3.34)**	**3.31**	**(−0.09 to 6.71)**	**1.83**	**(−1.64 to 5.31)**
Basal tone	A2	4.09	(0.27 to 7.91)	7.6	(3.90 to 11.30)	**2.12**	**(−1.72 to 5.97)**
Basal tone	A3	**8.61**	**(−7.32 to 24.54)**	**6.73**	**(−8.97 to 22.43)**	22.87	(6.82 to 38.91)
Basal tone	A4	9.08	(5.58 to 12.59)	8.47	(5.07 to 11.86)	6.53	(3.00 to 10.06)

PFMT: Pelvic floor muscle training; HE: Hypopressive exercise; PFMT+HE: Pelvic floor muscle training and hypopressive exercise; CI: Confidence Interval; PFDI-20: Pelvic floor Distress Inventory Short Form; POPDI: Pelvic organ prolapse distress inventory; UDI: Urinary distress inventory; CRADI: Colo-rectal-anal distress inventory; PFQ−7: Pelvic Floor Impact Questionnaire Short Form; POPIQ: Prolapse impact questionnaire; CRAIQ: Colo-rectal-anal impact questionnaire; UIQ: Urinary impact questionnaire. The mean and CI that did not show significant differences against A0 are highlighted in bold.

**Table 3:** Mean differences between intervention groups across 4 follow-up assessments.

Variable	(HE-G)–(PFMT-G)		(PFMT+HE-G)–(PFMT-G)	
	Mean Diff.	*p*-Value	Mean Diff.	*p*-Value
PFDI-20	−0.54	0.9326	11.67	0.0703
POPDI	−0.06	0.9785	3.83	0.0935
CRADI	−0.26	0.9040	3.09	0.1535
UDI	0.59	0.8503	4.82	0.1233
PFIQ-7	1.95	0.6675	**10.25**	**0.0248**
POPIQ	1.05	0.6175	**4.16**	**0.0485**
CRAIQ	1.64	0.2768	**3.86**	**0.0110**
UIQ	−0.84	0.7119	1.81	0.4260
Manometry	−1.44	0.5280	−0.04	0.9844
Dynamometry	−86.07	0.0887	−72.63	0.1481
Basal tone	1.04	0.7721	2.87	0.4333

PFMT-G: Pelvic floor muscle training group; HE-G: Hypopressive exercise group; PFMT+HE-G: Pelvic floor muscle training and hypopressive exercise group; diff: Difference; PFDI-20: Pelvic floor Distress Inventory Short Form; POPDI: Pelvic organ prolapse distress inventory; UDI: Urinary distress inventory; CRADI: Colo-rectal-anal distress inventory; PFIQ-7: Pelvic Floor Impact Questionnaire Short Form; POPIQ: Prolapse impact questionnaire; CRAIQ: Colo-rectal-anal impact questionnaire; UIQ: Urinary impact questionnaire. Average differences between groups that showed *p*-values < 0.05 are highlighted in bold.
